# Construction and validation of a measure of integrative well-being in seven languages: The Pemberton Happiness Index

**DOI:** 10.1186/1477-7525-11-66

**Published:** 2013-04-22

**Authors:** Gonzalo Hervás, Carmelo Vázquez

**Affiliations:** 1 School of Psychology, Complutense University, Madrid, Spain

**Keywords:** Psychological well-being, Life satisfaction, Quality of life, Measurement, Happiness, Cross-cultural

## Abstract

**Purpose:**

We introduce the Pemberton Happiness Index (PHI), a new integrative measure of well-being in seven languages, detailing the validation process and presenting psychometric data. The scale includes eleven items related to different domains of remembered well-being (general, hedonic, eudaimonic, and social well-being) and ten items related to experienced well-being (i.e., positive and negative emotional events that possibly happened the day before); the sum of these items produces a combined well-being index.

**Methods:**

A distinctive characteristic of this study is that to construct the scale, an initial pool of items, covering the remembered and experienced well-being domains, were subjected to a complete selection and validation process. These items were based on widely used scales (e.g., PANAS, Satisfaction With Life Scale, Subjective Happiness Scale, and Psychological Well-Being Scales). Both the initial items and reference scales were translated into seven languages and completed via Internet by participants (N = 4,052) aged 16 to 60 years from nine countries (Germany, India, Japan, Mexico, Russia, Spain, Sweden, Turkey, and USA).

**Results:**

Results from this initial validation study provided very good support for the psychometric properties of the PHI (i.e., internal consistency, a single-factor structure, and convergent and incremental validity).

**Conclusions:**

Given the PHI’s good psychometric properties, this simple and integrative index could be used as an instrument to monitor changes in well-being. We discuss the utility of this integrative index to explore well-being in individuals and communities.

## Background

Perceived well-being is of great importance for most human beings. Although there is cultural variation in the relevance assigned to happiness, a positive evaluation of one’s own life in conjunction with a state of positive emotions is universally valued as a significant component of a good life [1]. Given the centrality of well-being in people’s lives, its measurement is not only a way to assess human feelings and psychological capabilities, but is also a central aspect of comprehensive models of psychological health. Assessment of well-being is crucial for validating theories and models of well-being [2], and measuring the outcome of positive interventions, particularly clinical interventions [3,4]. Furthermore, short and valid measures of well-being are needed due to current interest in assessing well-being in large samples such as in national assessments. Thus, the assessment of well-being is essential for both experimental research and applied purposes.

Although there is an increasingly wide array of solid measures that independently cover different components of well-being (e.g., life satisfaction, positive emotions, psychological functioning, and social well-being) [2,5,6], there is still a need for brief, comprehensive measures that can be used to make rapid, reliable, and valid assessments [7]. Most existing measures only cover one well-being domain, even though well-being is a conceptually complex construct [8-11]. Furthermore, psychological well-being can be assessed using different timeframes of measurement (e.g., retrospective vs. momentary assessment), which adds an additional level of difficulty to the development of sound and comprehensive measures of this variable. We propose an integrative measure of well-being with the objective of providing an index that incorporates the most relevant domains of well-being indicated in the literature as well as different timeframes of assessment. In the next sections, we will explain our rationale for the new measure, describe its main components, which are derived from major theories of well-being [12,13], and justify these components.

### Hedonic and Eudaimonic Well-Being and Beyond

The concept and measurement of well-being have been studied from various perspectives. Most current authors emphasize the existence of two ways to conceptualize well-being: hedonia and eudaimonia [14]. Hedonic well-being underscores the importance of life satisfaction and affective components, whereas eudaimonic well-being is focused on optimal psychological functioning, which depends on self-fulfillment and includes the concepts of personal growth, purpose in life, and a sense of autonomy among others [9,13].

Recent research and theoretical elaboration on the eudaimonic perspective have provided sound arguments on ways to improve the design and measure of the well-being construct [15-19]. Authors from the eudaimonic tradition maintain that any well-being measure lacking eudaimonic components is incomplete [17,18]. In fact, people can feel happy and report experiencing happiness but lack other relevant features that characterize a psychologically healthy person [18]. An extreme example of this pattern is an individual suffering a manic episode who may report extremely positive feelings and high satisfaction with life without optimal daily functioning. Waterman [19] pointed out that “experiences of eudaimonia are always accompanied by experiences of hedonia, but…the reverse is not true” (p. 243). Nevertheless, it is important to note that life satisfaction and positive affect do not simply measure hedonic well-being [16]. These hedonic measures are strongly associated with eudaimonic experiences [18,19] and optimal functioning [20]. Thus, an adequate assessment of well-being requires assessing both affect, including evaluations of one’s own life, and positive (and negative) functioning [17,21].

Apart from the hedonic and eudaimonic distinction, some authors tried to expand this individualistic perspective by including societal aspects of subjective well-being. From attachment processes to later social bonds, social needs seem to be relevant across most developmental stages, and the depth of the social roots of human functioning and well-being is widely recognized (see [22]). However, social well-being goes beyond interpersonal relationships. In his influential proposal on optimal functioning and mental health, Keyes [23] points out that “individuals remain embedded in social structures and communities, and face countless social tasks and challenges” (p. 122). According to Keyes, the appraisal of one’s circumstances and functioning in society is also a necessary component of integrative models of well-being. As a result of the above arguments, we aimed to create a measure that includes an assessment of general (i.e., life satisfaction), hedonic (i.e., positive and negative affect), eudaimonic (i.e., optimal functioning), and social well-being (for a similar perspective, see [12]).

### The Remembered Versus Experienced Well-Being Controversy

There is general agreement on the need to take hedonic and eudaimonic aspects of well-being into account to adequately assess psychological well-being.^1^ There is less debate on the psychological factors affecting the self-assessment of well-being. Most available well-being instruments are focused on participants’ retrospective accounts or evaluations of their satisfaction level, happiness, or psychological functioning. These current assessment instruments mainly rely on “remembered well-being” [24], which is based upon participants’ memory and judgment of their lives. Although this means of assessment has proven useful, it is vulnerable to reporting biases from different sources (e.g., personality, culture, memory, and assessment conditions) [25-27].

A different approach to measurement focuses on “experienced well-being,” which assesses momentary affective states and people’s feelings in real time rather than relying on the memory of these states. These methods are inspired by the experience sampling method [6,28] where people are asked to note what they are doing and feeling in the very moment of the assessment. Daniel Kahneman and colleagues [29], for example, developed the Day Reconstruction Method (DRM), a procedure intended to reduce memory biases by asking people to reconstruct in detail how they spent their time and how they experienced the various activities and events of their lives within the past 24 hours (see a review of measures in [30]).

The assessment of experienced well-being may be relevant to the exploration of cross-cultural differences in well-being (see [31]). For instance, Oishi [32] compared well-being reports of Asian and European Americans and found that although there were no cultural differences in experienced well-being, European Americans reported a higher degree of well-being than Asians in retrospective global reports. Thus, the validity of cross-cultural research on well-being may improve by considering this dual perspective (i.e., remembered vs. experienced well-being). It should be clear that remembered and experienced well-being are not mutually exclusive but reflect complementary approaches to measure well-being.

### Characteristics of a Brief Measure of Integrative Well-Being

There are already relatively brief measures that tap into the well-being construct. Relevant measures include the Satisfaction With Life Scale (SWLS; [33]), Subjective Happiness Scale (SHS; [34]), Flourishing Scale (FS; [35]), the Mental Health Continuum-Short Form (MHC-SF; [36]), and Warwick-Edinburgh Mental Well-Being Scale (WEMWBS; [37]). Unfortunately, although these scales have proven useful in measuring some aspects of well-being, they fail to cover other relevant areas of psychological, social, and experienced well-being. Furthermore, most of these scales were created and validated in English, with some of them adapted for other languages and countries, such as the SWLS.

Thus, this study aimed to develop and validate a new measure of integrative well-being, the Pemberton Happiness Index (PHI), (a) that covers its different domains (i.e., general, hedonic, eudaimonic, and social), (b) that implements different approaches of assessment (i.e., remembered and experienced well-being), and (c) that is validated for a variety of languages and countries from its inception.

## Methods

### Sample

Data were collected from a sample of the general population (*N* = 4,407). Participants were from research panels (i.e., groups of people that agree to regularly participate in social surveys) of Millward Brown, a survey company that operates worldwide.^2^ We selected countries from diverse linguistic, religious, and cultural backgrounds. From Europe, we included Spain (Southern Europe), Germany (Central Europe), Sweden (Northern Europe), and Russia (Eastern Europe); from Asia, we chose Turkey (Western Asia), India (Central Asia), and Japan (Eastern Asia); from the Americas, we decided on the USA (predominantly English-speaking) and Mexico (predominantly Spanish-speaking). We also covered major religious traditions: Islam (Turkey), Catholic Christianity (Spain, Mexico, and the USA), Eastern Orthodox Christianity (Russia), Protestant Christianity (Germany and the USA), Hinduism (India), and Buddhism (Japan).

The composition of the samples was heterogeneous in terms of sex, socioeconomic status, and education level. Data from 355 participants (8.05%) were removed due to invalid responses (e.g., missing values or inconsistencies in information on age). The results reported here refer to the rest of the sample (*N* = 4,052; 2,041 male) with ages ranging from 16 to 60 years (*M* = 34.30 years, SD = 10.45). Regarding education level, 0.3% reported not having completed elementary school, 1.9% only completed elementary school, 8.9% completed middle school studies, 34.7% completed high school, and 54.2% obtained a college degree. Regarding living arrangements, 66.6% reported to be living with a partner. Table 1 shows the demographic characteristics of each country’s sample.

**Table 1:** Main sociodemographic characteristics of each country’s sample

	***n***	**Sex**	**Age**	**Education level:**	**Occupation:**	**Living with partner:**
		***Men n *(%)**	***M *(SD)**	***n *(%)**	***n *(%)**	***n *(%)**
				- No education	- Employed	
				- Elementary	- Unemployed	
				- Middle school	- Student	
				- High school	- Homemaker	
				- College	- Retired	
Germany	375	177 (47.2%)	38.05 (10.98)	2 (0.5%)	231 (61.6%)	272 (72.5%)
				38 (10.1%)	41 (10.9%)	
				125 (33.3%)	44 (11.7%)	
				38 (10.1%)	39 (10.4%)	
				91 (24.3%)	20 (5.3%)	
India	393	204 (51.9%)	32.18 (9.42)	0 (0%)	280 (71.2%)	260 (66.2%)
				4 (1.0%)	36 (9.6%)	
				19 (4.8%)	40 (10.2%)	
				38 (10.1%)	29 (7.4%)	
				367 (93.3%)	6 (1.5%)	
Japan	378	182 (48.1%)	39.42 (9.68)	0 (0%)	328 (86.8%)	108 (28.6%)
				0 (0%)	8 (2.1%)	
				170 (45.0%)	13 (3.4%)	
				38 (10.1%)	25 (6.6%)	
				192 (50.8%)	4 (1.1%)	
Mexico	373	198 (53.1%)	32.18 (10.68)	2 (0.5%)	243 (65.1%)	245 (65.7%)
				0 (0%)	49 (13.1%)	
				84 (22.5%)	56 (15.0%)	
				38 (10.1%)	20 (5.4%)	
				238 (65.4%)	5 (1.3%)	
Russia	402	190 (47.3%)	32.58 (11.01)	0 (0%)	286 (71.1%)	307 (76.4%)
				3 (0.7%)	36 (8.9%)	
				116 (28.9%)	34 (8.5%)	
				38 (10.1%)	35 (8.7%)	
				271 (67.5%)	11 (2.7%)	
Spain	990	505 (51.0%)	31.95 (8.90)	4 (0.4%)	587 (59.3%)	702 (70.9%)
				26 (2.6%)	175 (17.7%)	
				442 (44.6%)	174 (17.6%)	
				38 (10.1%)	38 (3.8%)	
				407 (41.1%)	16 (1.6%)	
Sweden	385	187 (48.6%)	37.96 (10.80)	0 (0%)	284 (73.8%)	283 (73.5%)
				2 (0.5%)	36 (9.4%)	
				223 (57.9%)	46 (11.9%)	
				38 (10.1%)	3 (0.8%)	
				128 (33.3%)	16 (4.2%)	
Turkey	371	207 (55.8%)	30.88 (8.64)	2 (0.5%)	217 (58.5%)	246 (66.3%)
				2 (0.5%)	65 (17.5%)	
				115 (31.0%)	62 (16.7%)	
				38 (10.1%)	13 (3.5%)	
				241 (65.0%)	14 (3.8%)	
USA	385	191 (49.6%)	37.36 (11.02)	1 (.3%)	248 (64.4%)	278 (72.2%)
				1 (.3%)	42 (10.9%)	
				112 (29.1%)	25 (6.5%)	
				38 (10.1%)	50 (13%)	
				257 (66.7%)	20 (5.2%)	

### Instruments

A) Integrative well-being: Scale development.

1. *Remembered well-being.* We generated items that had similar content to those included in well-known validated measures of well-being. After assessment by subject matter experts, an initial pool of 21 items was created to assess four domains of remembered well-being (i.e., general, eudaimonic, hedonic, and social well-being). Each domain or subdomain (eudaimonic well-being has six subdomains and hedonic well-being has two subdomains) consisted of at least two items. Item translation followed the standard guidelines of translation and back-translation procedures.^3^

a. *General well-being.* We included two items related to global satisfaction with life and one item of vitality as it is closely associated with eudaimonic functioning [38].

b. *Eudaimonic well-being.* Items covering optimal psychological functioning were derived from Ryff’s psychological well-being model [13]. We put together a list of 12 items addressing the following subdomains that are equivalent to Ryff’s six areas of psychological well-being: life meaning, self-acceptance, personal growth, relatedness, perceived control, and autonomy.

c. *Hedonic well-being.* Affective state was assessed with items reflecting the frequency of positive and negative affect in daily life with two items for each affect type.

d. *Social well-being*. Although there are several components of social well-being (see [23,39]), we selected two items that tap into the global feeling of living in a society that promotes optimal psychological functioning.

Participants were asked to rate each of the 21 statements using a scale from 0 (*fully disagree*) to 10 (*fully agree*). (See Appendix for the English version).

2. *Experienced well-being.* We created a list of 16 items related to specific experiences. To construct this list, we followed an approach similar to the one used in the Gallup-Healthways Well-Being Index [40] which in turn was based on the Day Reconstruction Method [29]. Participants were presented with eight common positive events (e.g., “I hugged someone”) and eight negative ones (e.g., “I had an argument with someone”) that can be experienced by virtually anyone on a given day in different cultures. Participants were simply asked to state whether these events occurred the day before. The final 10 items and the response format are presented in the Appendix.

B) Validation measures.

In addition to our initial pool of 37 items specifically generated for our scale, the Internet-based survey also included a battery of highly validated well-being measures. These measures were used as criteria to validate the items of our scale which were chosen to produce the best validity results for all languages and countries. We chose convergent validity as the main criterion so that final items (not only total scores, which are typically used in the validation of similar scales) were those that showed the highest mean correlations with their respective validation measures across countries. The following instruments were included to validate the items:

1. *Remembered well-being.*

a. *General well-being.* We used three measures to validate the items examining general well-being: the SWLS [33], SHS [34], and Satisfaction With Domains of Life (SWDL). The SWLS includes five items to assess the cognitive component of life satisfaction (e.g., “I am satisfied with my life”); it is the most common scale used to assess global satisfaction with life and has been implemented in several languages and cultures, providing good psychometric indices [41]. Similar to the SWLS, the SHS is a four-item scale that assesses a general appreciation for life and personal feelings of happiness; it has been validated in several countries using different types of samples and results have indicated that the SHS has high internal consistency and sound test-retest reliability. For the SWDL, following published literature on the assessment of life satisfaction [33,42,43], we selected 12 different domains of life (e.g., relationships, family, friends, health, income, city, and country); participants were asked to rate their responses on a scale from 0 (*very dissatisfied*) to 10 (*very satisfied*), and a total score of satisfaction with the domains of life was calculated by summing up all the items.

b. *Eudaimonic well-being.* We used Ryff’s Scales of Psychological Well-Being (SPWB; [13]) to validate the items measuring eudaimonic well-being. Although there are several versions of the scale, we used the 39-item version [44,45], a questionnaire that covers the six areas of psychological well-being proposed in Ryff’s model with six to eight items per area (environmental control, autonomy, positive relationships, purpose in life, personal growth, and self-acceptance).

c. *Hedonic well-being.* We used the PANAS [46] to validate the items associated with hedonic well-being. This 20-item scale, assessing 10 positive and 10 negative emotions, is the most commonly used scale to assess positive and negative affect. It has been adapted for use in several languages and cultures [47].

d. *Social well-being.* We used the SWDL item that assesses satisfaction with one’s own country as well as total scores from the SWLS and SHS to validate the items related to global social well-being.

2. *Experienced well-being.* Here, we sought to determine the participant’s satisfaction with the previous day. To validate our 10-item measure of experienced well-being (i.e., experiences that occurred the day before), we included a question aimed at assessing the participant’s overall well-being experienced the day before (i.e., “How did you feel yesterday?”) rated on a Likert scale from 0 (*very badly*) to 4 (*great*).

Aside from this set of questionnaires addressing remembered and experienced well-being, we included two additional questions on health issues in the web-based interview for further validation purposes. As perceived health is a consistent proxy of happiness and well-being [48,49], participants were asked to rate their health (i.e., “How, in general, would you rate your health at this moment?”) on a Likert scale from 0 (*very poor health*) to 10 (*very good health*). In a second question, they were asked to rate their sleep quality (i.e., “How much rest do you get when you sleep?”), which has also been linked to subjective well-being [50], on a Likert scale from 0 (*none at all*) to 10 (*total rest*).

### Procedure

Data collection occurred between December 1 and 15, 2009. Using the Computer-Assisted Web Interviewing (CAWI) technique, all questionnaires were programmed into a web-based application with the content translated into seven different languages, and data were collected in an online database. Participants were invited via email and received a small incentive for their participation (i.e., they received points, which each had a value of $5, that could be accumulated and exchanged for an object from a list of goods). The percentage of panelists who initially agreed to participate but later declined to do so was 21.7%. Finally, 26.4% of the panelists initially entered the study were screened out early on because of inability to fulfill the panel quota requirements (sex, age, and location). The average time to answer the complete questionnaire was 21.0 (± 4.09) minutes.

### Analytic strategy

Our aim was to create a scale based on items from the initial pool that maximized overall convergent validity across countries. First, each of the 21 initial remembered well-being items was correlated with its respective validation criterion (e.g., positive affect items were correlated with the positive subscale of the PANAS). Items showing the highest mean correlation across countries were chosen for inclusion in the final scale. A similar procedure was followed to select the experienced well-being items. An initial pool of 16 common experiences with potential to have an emotional impact (8 positive, 8 negative) was created by the authors. The validation criterion for this pool was participants’ evaluation of their overall satisfaction with the day before. Given the dichotomous nature of the experienced well-being items (i.e., yes/no responses), final item selection was based on Cramer’s V (a commonly-used measure of association for the chi-squared test). The ten experienced well-being items (5 positive, 5 negative) with the highest effect sizes across countries were chosen for inclusion in the final scale. Reliability was examined by internal consistency (Cronbach’s alpha).

Structural validity was assessed using principal components factor analysis, and the number of factors was determined through the Velicer's minimum average partial test [51,52]. Incremental validity was tested with a series of regression analyses examining the predictive value of our scale above and beyond the SHS, SWLS, six SPWB subscales, and two PANAS subscales, using subjective health and sleep quality as the criteria.

## Results

### Item Selection for Remembered Well-Being

To reach a set of items with the highest convergent validity for the whole set of countries, correlations between each of the 21 initial items and their respective comparison scales were calculated for each country. Items that showed the highest overall mean correlations with their corresponding criteria were chosen to be included in the scale. Overall correlations for the final selected items are reported in Table 2.

**Table 2:** **Mean correlations across countries for remembered well-being items (excluding experienced well-being) in the Pemberton Happiness Index (see Appendix)**

**Domains and subdomains**	**Reference scales**	**Mean correlation**
*General well-being*	SWLS; SHS; SWDL	.69 (Item r1)
		.48 (Item r2)
*Eudaimonic well-being*		
Life meaning	SPWB: Purpose in life	.60 (Item r3)
Self-acceptance	SPWB: Self-acceptance	.67 (Item r4)
Personal growth	SPWB: Personal growth	.50 (Item r5)
Relatedness	SPWB: Positive relationships	.48 (Item r6)
Competence	SPWB: Environmental control	.57 (Item r7)
Autonomy	SPWB: Autonomy	.41 (Item r8)
*Hedonic well-being*		
Positive affect	PANAS: Positive affect	.50 (Item r9)
Negative affect	PANAS: Negative affect	.47 (Item r10)
*Social well-being*	SWDL: Satisfaction With Country; SWLS; SHS	.42 (Item r11)

*Note.* Items are ordered in the table as they appear in the final scale (see Appendix). SWLS = Satisfaction With Life Scale; SHS = Subjective Happiness Scale; SWDL = Satisfaction With Domains of Life; SPWB = Ryff’s Scales of Psychological Well-Being; PANAS = Positive and Negative Affect Schedule.

### Item Selection for Experienced Well-Being

Items were selected so that the final scale presented the highest convergent validity for all countries. For these analyses, we employed the item assessing overall satisfaction with the day before as the validation criterion. Chi-square analyses were conducted between each item and the criterion. Items that showed the highest Cramer’s V values were selected for inclusion in the final scale (Table 3).

**Table 3:** Mean association (Cramer’s V) across countries between satisfaction with the day before and the 10 items on experienced well-being (five negative and five positive)

**Experienced well-being**	**Reference item**	**Item**	**Mean Cramer’s V**
Positive experiences	Satisfaction with the day before	Something I did made me proud	.33
		I did something fun with someone	.36
		I did something I really enjoy doing	.38
		I learned something interesting	.28
		I gave myself a treat	.28
Negative experiences	Satisfaction with the day before	At times, I felt overwhelmed	.29
		I was bored for a lot of the time	.32
		I was worried about personal matters	.33
		Things happened that made me really angry	.33
		I felt disrespected by someone	.28

### Calculating Experienced Well-Being

The Pemberton Happiness Index (PHI) was designed as a brief measurement of overall well-being that includes both remembered and experienced well-being. Although data for these two types of well-being can be separately obtained in the PHI, a procedure was designed to provide a combined well-being index. This index is the sum of positive experiences (each counted as “1”) and absences of negative experiences (each counted as “1”) of the day before. With this procedure, a single overall score of experienced well-being can be calculated, ranging from 0 to 10 similarly to the items from the remembered well-being scale. Other researchers used this method in the past to reach a single score based on positive and negative experiences of the day before [53].

In our nine samples, correlations between remembered and experienced well-being indices ranged from .46 to .61 (all *p*’s < .001) with a mean of .53. After correcting for unreliability, correlations ranged from .64 to .76 with a mean of .69. Overall, these results suggest that the two scales are measuring related but different constructs.

Thus, the PHI index incorporates two components: (a) an 11-item measure that includes general, eudaimonic, hedonic, and social well-being rated on a scale from 0 to 10 and (b) a single score that results from the combination of positive and negative experiences from the day before also on a scale from 0 to 10.

### The Pemberton Happiness Index

Table 4 shows the final items empirically selected for the PHI. It contains 11 items related to different domains of remembered well-being (i.e., general, eudaimonic, hedonic, and social well-being) and 10 items related to experienced well-being, which can be transformed into a single well-being index using the same scale as the other 11 items.

**Table 4:** Final items for remembered and experienced well-being

**Domains and subdomains**	**Item content**
**Remembered well-being**	
*General well-being*	I am very satisfied with my life
	I have the energy to accomplish my daily tasks
*Eudaimonic well-being*	
Life meaning	I think my life is useful and worthwhile
Self-acceptance	I am satisfied with myself
Personal growth	My life is full of learning experiences and challenges that make me grow
Relatedness	I feel very connected to the people around me
Perceived control	I feel able to solve the majority of my daily problems
Autonomy	I think that I can be myself on the important things
*Hedonic well-being*	
Positive affect	I enjoy a lot of little things every day
Negative affect	I have a lot of bad moments in my daily life
*Social well-being*	I think that I live in a society that lets me fully realize my potential
**Experienced well-being**	
* Positive experiences*	Something I did made me proud
	I did something fun with someone
	I did something I really enjoy doing
	I learned something interesting
	I gave myself a treat
* Negative experiences*	At times, I felt overwhelmed
	I was bored for a lot of the time
	I was worried about personal matters
	Things happened that made me really angry
	I felt disrespected by someone

For calculating the final score in the remembered well-being scale we divided each individual’s sum of raw scores by eleven (i.e., the number of items of the scale), which provides a mean score from 0 to 10. To calculate the overall PHI index, which included remembered and experienced well-being, we sum the individuals’ scores of the 11 items related to remembered well-being plus the sum of scores on the experienced well-being; the total sum is then divided by 12, so the resulting PHI total mean score also ranges from 0 to 10. Means and standard deviations of the PHI are reported in Table 5. No significant associations between the PHI total score and age or sex were found for any country.

**Table 5:** Means (M), standard deviations (SD), of remembered and experienced well-being

	***n***	**Remembered well-being (11 items)**		**Experienced well-being (1 item)**	
		***M***	***SD***	***M***	***SD***
Germany	375	6.55	2.00	6.22	2.34
India	393	7.23	1.61	6.33	2.17
Japan	378	4.92	1.66	5.38	2.06
Mexico	373	7.91	1.80	7.42	2.20
Russia	402	6.53	1.83	6.38	2.05
Spain	990	6.92	1.71	6.66	2.17
Sweden	385	6.76	1.97	6.64	2.09
Turkey	371	6.08	1.68	5.50	2.66
USA	385	6.93	1.95	6.32	2.49

### Internal Consistency

We calculated Cronbach’s alpha for both the 11-item scale (excluding experienced well-being) and the expanded scale (including experienced well-being) for each country (see Table 6). Adding the experienced well-being score based on positive and negative experiences from the day before did not change the consistent pattern of high internal reliability. In all countries, the internal consistency of the scales (in both the 11-item version and 11+1-item version) was above .89 with the exception of the Turkey sample (Cronbach’s alpha from .82 to .83).

**Table 6:** Cronbach’s alpha (α) for the 11-item index (excluding experienced well-being) and 11+1-item index (including experienced well-being) of the Pemberton Happiness Index

	**α (11 items)**	**α (11+1 items)**	**% variance**
Germany	.93	.93	59.56
India	.89	.89	54.56
Japan	.92	.93	57.49
Mexico	.92	.92	59.38
Russia	.89	.90	49,61
Spain	.91	.92	56.08
Sweden	.92	.92	59.06
Turkey	.82	.84	41.13
USA	.93	.93	60.95

*Note.* The table also shows the percentage of variance explained by a unifactorial solution for the 11-item version of the Pemberton Happiness Index.

### Inter-Item and Item-Total Correlations

Mean inter-item correlations of the PHI within each country ranged from .31 (Turkey sample) to .56 (USA sample). According to Briggs and Cheek ([54], p. 115), mean inter-item correlations between .2 and .4 indicate an optimal level of homogeneity.

To further explore the consistency of the PHI, we calculated item-total correlations of the PHI for each country. Mean item-total PHI score correlations ranged from .61 (Turkey sample) to .77 (German and USA samples). Previous literature has suggested that item-total correlations, in sound psychometric instruments, should be higher than .30 [55].

### Convergent Validity

The PHI showed a consistent pattern of correlations with the scales included in this study that covered different aspects of well-being. As Table 7 shows, all but one correlation between the PHI total score and validation scales were positive. The exception was the PANAS negative, which showed a consistent pattern of negative correlations as expected.

**Table 7:** Correlations between the Pemberton Happiness Index and the study variables

	**Pemberton Happiness Index (11+1)**								
	**Germany (*n *= 375)**	**India (*n *= 393)**	**Japan (*n *= 378)**	**Mexico (*n *= 373)**	**Russia (*n *= 402)**	**Spain (*n *= 990)**	**Sweden (*n *= 385)**	**Turkey (*n *= 371)**	**USA (*n *= 385)**
SWLS	.71	.59	.66	.50	.61	.52	.77	.51	.66
SHS	.79	.69	.77	.73	.75	.69	.80	.71	.83
PANAS-P	.64	.60	.61	.75	.46	.70	.73	.45	.67
PANAS-N	-.57	-.33	-.46	-.54	-.48	-.43	-.60	-.45	-.48
SPWB									
Self-acceptance	.78	.65	.78	.72	.72	.74	.83	.69	.78
Positive relations	.55	.44	.62	.51	.45	.52	.67	.43	.62
Autonomy	.25	.31	.42	.46	.32	.39	.41	.25	.32
Enviromental control	.76	.61	.71	.68	.71	.71	.81	.62	.81
Personal growth	.48	.41	.63	.55	.41	.49	.52	.44	.50
Purpose in life	.73	.66	.74	.68	.71	.68	.76	.61	.76
SWDL	.72	.76	.78	.75	.78	.76	.81	.75	.81

*Note.* All correlations were significant (*p* < .001). SWLS = Satisfaction with Life Scale; SHS = Subjective Happiness Scale; PANAS = Positive and Negative Affect Schedules (P = Positive; N = Negative); SPWB = Ryff’s Scales of Psychological Well-Being (39-item version); SWDL = Satisfaction With Domains of Life.

### Structural Validity

We expected a unifactorial model to fit our scale well (excluding the experienced well-being component). This expectation is based on previous research showing that when hedonic, eudaimonic, and social well-being are evaluated together with different scales, a model with a single higher-order factor (i.e., integrative well-being) and 14 facets (positive and negative affect, satisfaction with life, etc.) fits the data adequately. Furthermore, this model does not differ much from one with three different higher-order factors (i.e., hedonic, eudaimonic, and social well-being) [56]. Since we implemented a reduced set of 11 items to measure all components of well-being, it is reasonable to expect that only one factor will emerge (i.e., integrative well-being). Moreover, other brief scales tapping into different components of well-being have been unifactorial [37].

Consequently, for each country, a principal components analysis was conducted. It was found that a single factor (with eigenvalues < 1) consistently emerged, explaining a substantial portion of variance for most countries. The only exception was the Indian sample in which we found a second factor that included only the inverse item (i.e., item r10 negative affect). Percent variance explained for each sample (i.e., when one-factor is retained) is shown in Table 5. Using a more reliable method, the Velicer test, we found that a one-factor solution was also recommended for all countries.

### Incremental Validity

To evaluate the incremental validity of the PHI for the whole sample compared to other widely used well-being scales, we conducted a series of separate regression analyses, employing sleep quality and perceived health as criteria because they are often considered adequate proxies of well-being [20,57]. The PHI predicted sleep quality over and above the SHS (ΔR^2^ = .029, *p* < .001), the SWLS (ΔR^2^ = .056, *p* < .001), the six SPWB (ΔR^2^ = .052, *p* < .001), and the two PANAS (ΔR^2^ = .040, *p* < .001). Furthermore, we found that our scale predicted perceived health over and above the SHS (ΔR^2^ = .042, *p* < .001), the SWLS (ΔR^2^ = .088, *p* < .001), the six SPWB (ΔR^2^ = .052, *p* < .001), and the two PANAS (ΔR^2^ = .051, *p* < .001).

## Discussion

The PHI is based on the conceptual integration of current approaches to defining and measuring well-being. Our results suggest that the PHI is a consistent and valid instrument to provide an index of well-being.

The PHI has some noteworthy advantages relative to previous composite indices of well-being. From a conceptual point of view, this is the first instrument that attempts to cover the main domains of well-being described in current theories and research in the area. The PHI was designed taking into account prevailing controversies on the eudaimonic versus hedonic distinction and the remembered versus experienced approach. All these aspects of well-being are relevant, and integrative measures should be aware of the complexity of the well-being construct [18,31].

Compared with other recently published brief well-being instruments, the PHI indeed encompasses a more thorough sense of the construct. For example, the MHC-SF [36] measures hedonic, eudaimonic, and social well-being but does not include experienced well-being. Also, the WEMWBS [37], in both its 7-item and 14-item versions, comprises positively phrased statements covering both hedonic and eudaimonic aspects of well-being, including positive affect, satisfying interpersonal relationships, and positive functioning. However, it does not include a specific item covering life satisfaction and does not cover experienced well-being or social well-being. Another recent brief index of well-being is the FS [35], which “was designed to measure social-psychological prosperity [and] to complement existing measures of subjective well-being” (p. 144). The FS is an eight-item scale that aims to provide a single index covering aspects of social capital, flow, social relationships, and a general sense of psychological prosperity (i.e., only partially covers some eudaimonic aspects of well-being). The authors who developed the FS used several well-known, validated instruments like the ones used in our study (i.e., the SHS, SWLS, PANAS, and SPWB) to assess the FS’ convergence validity. Yet, the scale was initially validated using only samples of university students.

Unlike other scales, the PHI includes a experience well-being section. Our data support that remembered and experienced well-being are related but different constructs. It is important to note that the experienced well-being section can be included or not in the index depending on the needs of the researcher and the characteristics of the sample. For example, in very small samples as well as for individual assessments, data from specific experiences that happened the day before, which is measured in the experienced well-being section, could be biased due to non-representative events (e.g., a sudden stressful event) that occurred the day before. On the contrary, larger samples make these random effects irrelevant as positive and negative non-representative events tend to equally happen compensating this potential source of bias. In sum, our index includes two separate scales assessing remembered and experienced well-being. Although these two subscales can be used separately, we suggest using them jointly especially when assessing the well-being of a community or a large sample.

Moreover, beyond these conceptual aspects, the PHI has distinctive methodological features. All the items included in the scale were empirically selected after being contrasted with widely used measures of each well-being domain. The study sample was also larger and more culturally diverse than in previous initial validation studies of similar brief scales.

Most notably, no other brief scale allows the use of each of its items as indices of different well-being domains. In designing and validating the PHI, we aimed for it to provide both a composite measure and individual measures of the different facets of well-being. As such, the PHI can be a valuable diagnostic tool when its items are used as individual and independent indicators. Although using a single-item scale implies diminished psychometric properties, it has been noted that this allows for an efficient assessment when needed [58]. Moreover, our data showed that the capacity to detect differences among countries was equal or even slightly better when measured by our single-item subscales compared with other larger scales. This strength could be due, at least in part, to the use of an 11-point Likert scale. Although there is no consensus on the effect of an increase in the number of categories of a scale, it may foster variability. While some authors defend the idea of using no more than 7 categories [59], others favor the idea of using more categories [60]. In their meta-analysis, Saris and Gallhofer [61] conclude that using an 11-point scale does not harm the reliability and validity of an instrument. According to this meta-analysis, it is more relevant to use a scale with a middle point and with clear, short labels for the two extremes. Both requirements are fulfilled in our scale. Moreover, an empirical study examining the role of different response options in the context of assessing subjective quality of life concluded that using a 10-point scale yielded better outcomes [62].

Finally, contrary to previous scales, we developed and validated the PHI for seven languages and nine countries, which increases its cross-cultural value. Although some versions may be refined in the future, this initial validation tentatively allows the use of the PHI in different countries and cultures. And, more relevant for cross-cultural research, the items included in the scale were chosen so that they maximize the convergent validity for the whole set of countries.

Our data support the fact that the PHI presents good psychometric properties. Given the nature of the measure, it is not surprising that the PHI positively correlates with validated measures of life satisfaction, positive affect, and eudaimonic well-being, and negatively correlates with negative affect. This correlation pattern suggests that the PHI reliably measures different aspects of well-being. The internal consistency of the PHI was very good for all language versions and inter-item correlations were consistently high. Some authors have warned against high homogeneity as it may indicate that several items have been paraphrased [63]; however, this is not our case. Instead, each of the items assesses a totally different dimension of well-being. Thus, the high homogeneity within the PHI may suggest the existence of a single construct. In fact, our principal components analysis indicates that the PHI has a single structure even as it integrates a complex conception of well-being involving its different aspects. Future research should confirm the factor structure in another sample (i.e., confirmatory factor analysis) and then verify the presence of measurement invariance across different language versions of the index [64].

We also acknowledge some limitations of this initial study. First, it would have been ideal to start with a larger pool of initial items. Due to the difficulties and costs of working with seven versions of the scale, we tried to select the best items before starting the translation and validation processes. Second, our study was conducted online. Although research has found that web-based surveys provide results as valid as those gathered with more traditional methods [65], we cannot completely rule out the existence of biases affecting web-based surveys that may not have yet been discovered. Nevertheless, our pattern of results is robust (in terms of internal consistency and validation indices of the PHI), which counters this possibility. Third, it is possible that some shared common variance is due to the assessment method. Finally, despite the effort to include a wide range of countries and languages, we were not able to incorporate other important languages (e.g., French and Chinese) and geographical areas (e.g., Africa) into the study. Furthermore, although the sample composition was larger and more heterogeneous than the samples used to validate similar instruments, the data in this study should not be considered representative of each country. Even so, the cross-national consistency of the results and the good psychometric properties of the PHI in all languages and participating nations are still noteworthy.

We are aware that the use of a self-report retrospective approach, such as the one used in the PHI and the Gallup study [66], does not completely preclude memory and judgmental biases when assessing experienced well-being. A better measurement option would involve costly procedures, as in the original DRM [29], or a sophisticated experience sampling method [6,67]. Yet, an alternative and simpler measure of experienced well-being, such as the one used in our study, has shown to provide information different from typical remembered well-being measures [68].

## Conclusion

Future studies, specifically conducted within each country, should also analyze the PHI’s sensitivity to change as well as the temporal stability of the index.^4^ It is important to know if changes in personal, psychological, or material circumstances significantly affect PHI scores. If so, the PHI could be used as a valid instrument to monitor changes in well-being. It will also be relevant to determine how these new indices are associated with non-self-report assessments of the same concepts by obtaining reports from informants or recording actual behaviors for example (see [31]).

The PHI can be considered a broad measure of well-being. There is some debate about the use of broad or narrow psychological dimensions to predict specific behaviors. Some authors have argued that broader measures are better predictors because they have greater reliability than narrower measures, and the variance in outcomes associated with broad factors generalizes across situations [69,70]. On the contrary, others support the idea that specific traits or psychological dimensions more efficiently predict specific behaviors or outcomes [71]. Although our results show that the PHI is associated with specific conditions (e.g., sleep problems), future research should address the issue of its ability to predict broad versus specific outcomes.

We fully agree with Helliwell and Barrington-Leigh’s [72] contention that although new indicators are welcome in research on well-being, they depend on the items included and the use of a priori weights on its components. Thus, as these authors point out, “the resulting indicators necessarily reflect the preferences of the designer of the index” (p. 736). There is no doubt that other indices can and will be developed; the utility of these new measures ultimately depends upon their ability to reliably and efficiently identify and predict differences across individuals or populations. To summarize, our aim was to develop and validate a new and comprehensive measure of well-being in different languages and cultures. We hope that this new instrument will contribute to advancement in the complex task of measuring well-being.

## Endnotes

^1^In this article, we use the term “psychological well-being” as the experience of a stable, global, and deep sense of well-being, the latent variable associated with our perspective of integrative well-being. Note that in some contexts, psychological well-being is used to name one of the modern eudaimonic theories formulated by Ryff [10].

^2^ Online surveys have been found to be better than other survey methods (i.e., telephone surveys) in some aspects such as less item nonresponse, and worse in other aspects such as response rate and item differentiation [73]. Low response rate in combination with low Internet penetration (i.e., in India, Mexico, and Turkey) can threaten representativeness of the sample, biasing toward wealthier populations. However, this is not a serious limitation in our case for several reasons: (a) The panel selected individuals until achieving a sample representative of the country in terms of sex, age, and location; (b) Representativeness is an important issue to reliably assess the well-being of a nation but is not crucial at all for validating a new measure; (c) The final sample for each country is much more diverse than opportunistic samples (e.g., university students) used in most published studies that initially validate psychological measures.

^3^ We followed the criteria described by Hambleton and Patsula [74] who suggested that a rigorous instrument adaptation process should involve at least three steps: (a) translating the test from a source to target language, (b) translating the test back into the source language (back translation), and (c) using independent teams of qualified translators to review the original, back-translated, and target language versions of the instrument to examine equivalence and resolve discrepancies.

^4^ Initial results from a different study, using a university sample (*n* = 53), suggest that 1-month test-retest reliability is high (*r* = .88).

## Appendix

The Pemberton Happiness Index

### Section A

Using the following scale from 0 to 10, with 0 being total disagreement and 10 being total agreement, please rate the extent to which you agree with the following statements.

Totally disagree 0 1 2 3 4 5 6 7 8 9 10 Totally agree

(r1) I am very satisfied with my life

(r2) I have the energy to accomplish my daily tasks

(r3) I think my life is useful and worthwhile

(r4) I am satisfied with myself

(r5) My life is full of learning experiences and challenges that make me grow

(r6) I feel very connected to the people around me

(r7) I feel able to solve the majority of my daily problems

(r8) I think that I can be myself on the important things

(r9) I enjoy a lot of little things every day

(r10) I have a lot of bad moments in my daily life*

(r11) I think that I live in a society that lets me fully realize my potential

*Note.* Items 1–2: general well-being; items 3–8: eudaimonic well-being; items 9–10: hedonic well-being; item 11: social well-being. * = Reverse scoring.

### Section B

Please mark which of the following happened to you *yesterday* (YES / NO):

(e1) Something I did made me proud

(e2) At times, I felt overwhelmed

(e3) I did something fun with someone

(e4) I was bored for a lot of the time

(e5) I did something I really enjoy doing

(e6) I was worried about personal matters

(e7) I learned something interesting

(e8) I gave myself a treat

(e9) Things happened that made me really angry

(e10) I felt disrespected by someone

*Note.* Items 1, 3, 5, 7, and 8 are positive experiences; items 2, 4, 6, 9, and 10 are negative experiences. These ten items can be converted into a single score from 0 (zero positive experiences and 5 negative experiences) to 10 (five positive experiences and no negative experiences). See Methods section for further details. Versions of the scale in different languages are available in http://www.pembertonindex.com.

## Competing interests

No conflict of interest is known.

## Authors’ contributions

Both authors were equally involved in planning the study, drafting, and amending the manuscript. Both authors have read and approved the final manuscript.

## Permission to Use

The Pemberton Happiness Index (PHI) is in the public domain (not copyrighted) and therefore researchers and practitioners are free to use it without permission or charge as long as they give credit to its authorship (See further details in http://www.pembertonindex.com).
